# Impact of Pharmacist-Led Implementation of a Community Hospital-Based Outpatient Parenteral Antimicrobial Therapy on Clinical Outcomes in Thailand

**DOI:** 10.3390/antibiotics11060760

**Published:** 2022-06-02

**Authors:** Teeranuch Thomnoi, Virunya Komenkul, Abhisit Prawang, Wichai Santimaleeworagun

**Affiliations:** 1 Department of Pharmacy, Faculty of Pharmacy, Silpakorn University, Nakhon Pathom 73000, Thailand; nuchrx@hotmail.com (T.T.); komenkul_v@silpakorn.edu (V.K.); 2 Pharmaceutical Initiative for Resistant Bacteria and Infectious Diseases Working Group (PIRBIG), Faculty of Pharmacy, Silpakorn University, Nakhon Pathom 73000, Thailand; 3 Pharmacy Unit, Khlong Luang Hospital, Pathum Thani 12120, Thailand; 4 Pharmacy Practice, College of Pharmacy, Rangsit University, Pathum Thani 12000, Thailand; apisit.p@rsu.ac.th

**Keywords:** antimicrobial stewardship, drug-related problem, outpatient parenteral antimicrobial therapy, pharmacist intervention

## Abstract

Few studies have analyzed community hospital-based parenteral anti-infective therapy (CohPAT). We aimed to assess the clinical impact of a pharmacist-led implementation of a clinical practice guideline (CPG) for CohPAT, and to determine the pharmacist’s role in CohPAT medication management. The prospective-period patients (post-implementation group) were compared with the historical control-period patients (pre-implementation group) for receiving a continuous antimicrobial parenteral injection. A CPG was used for laboratory testing for efficacy and safety, the monitoring of adverse drug events during admission, microbiology results coordination, and dosage adjustment. For any antimicrobial drug-related problems, the pharmacist consulted with the clinicians. Over 14 months, 50 participants were included in each group. In the pre-implementation period, 7 (14%) and 4 (8%) out of 50 patients received an inappropriate dosage and nonlaboratory monitoring for dose adjustment, respectively. The patients received the proper dosage of antimicrobial agents, which increased significantly from 78% pre- to 100% post-implementation (*p* = 0.000). The pharmacist’s interventions during the prospective-period were completely accepted by the clinicians, and significantly greater laboratory monitoring complying with CPG was given to the postimplementation group than the pre-implementation group (100% vs. 60%; *p* = 0.000). Significantly less patients with unfavorable outcomes (failure or in-hospital mortality) were observed in the post-implementation than in the pre-implementation (6% vs. 26%; *p* = 0.006) group. For the logistic regression analysis, lower respiratory infection (adjusted OR, aOR 3.68; 95%CI 1.13–12.06) and the post-implementation period (aOR 0.21; 95%CI 0.06–0.83) were significant risk factors that were associated with unfavorable outcomes. Given the better clinical outcomes and the improved quality of septic patient care observed after implementation, pharmacist-led implementation should be adopted in healthcare settings.

## 1. Introduction

Infectious diseases due to multi-drug resistant (MDR) pathogens remain a major public health problem worldwide [1]. Over the last several years, the antimicrobial resistance of bacteria has become one of the leading causes of mortality [2]. With an estimated 19,122 deaths in 2010 due to multidrug resistant infections in Thailand [3], approximately 700,000 people die worldwide from microbial resistant infections every year, which has an adverse economic impact worldwide [4]. In 2017, the World Health Organization (WHO) launched the global priority list of antimicrobial-resistant bacteria as a guide for researchers seeking new treatments [1]. Unfortunately, the treatment regimens against MDR-bacterial infections remain limited. The restricted options available have led to the overuse and misuse of antibiotics, and insufficient numbers of newly developed antibiotics in the pipeline. Health care professionals have been forced to wisely use available antibiotic agents [5]. Therefore, the development or optimization of antibiotics is needed to effectively treat MDR-bacterial infections.

The Antimicrobial Stewardship Program (ASP) is a coordinated program that promotes the appropriate use of antimicrobials, improves patient outcomes, reduces the rate of resistant pathogens, decreases the spread of infections, and reduces medical costs [6]. The emphasis is placed upon appropriate antimicrobial selection, dosing regimens, routes of administration, and durations of antimicrobial therapy [7]. ASP is a collaborative and multidisciplinary approach consisting of physicians, clinical pharmacists, clinical microbiologists, information system specialists, and infection control specialists, with accountability through a single physician or pharmacist leader for program management and outcomes [6,7].

Outpatient Parenteral Antimicrobial Therapy (OPAT) is one of the ASP strategies that provides long-term intravenous antimicrobial treatment without the longer duration of a hospital stay. OPAT is used by various health care providers, such as outpatient departments, emergency departments, and nursing care or rehabilitation centers [8]. OPAT has been shown to be safe, clinically effective, and cost effective. Chapman et al. examined the clinical efficacy and cost effectiveness of an OPAT service involving an outpatient infusion center and the patient/caregiver administration models of service delivery. The researchers found that OPAT led to an 87% cure or improvement rate upon the completion of intravenous therapy, with a re-admission rate of 6.3%, and OPAT costs only around 40% of the equivalent inpatient costs in a hospital setting [9]. Similarly, Durojaiye et al. reported an 88% successful outcome (cure or improvement) rate and a re-admission rate of 7%. The OPAT cost was only 15% of the inpatient costs for an infectious disease unit [10]. Therefore, OPAT was found to be safe, clinically efficacious, and acceptable for treating a wide range of infections, with high levels of patient satisfaction and substantial cost savings.

In Thailand, OPAT practice has been considered or implemented via the provision of continuous antimicrobial parenteral injections in an infusion center established by hospitals admitting infectious patients [11], or at community hospitals as a community hospital-based parenteral anti-infective therapy (CohPAT) [12]. Unfortunately, only one report has considered the clinical outcomes of CohPAT [12]. Pharmacists, as members of ASP teams, could significantly improve patient care in CohPAT. Most notably, pharmacists have demonstrated positive contributions in the areas of clinical monitoring, antimicrobial dosing adjustment, and adverse drug monitoring among patients receiving OPAT and medication reconciliation during care transitions [13] Pharmacists play an important role in CohPAT, and they may have a positive effect on clinical outcomes.

The study aimed to examine the clinical impacts of a pharmacist-led implementation of the clinical practice guidelines (CPG) of CohPAT. Moreover, we simultaneousl determined the clinical pharmacist’s role in medication management within CohPAT.

## 2. Material and Methods

### 2.1. Participants

We conducted a pre- and post-implementation study with prospective period from October 2020 through May 2021, including a historical control group from March through August 2020. The inclusion criteria in this study were: (1) ≥18 years of age, and (2) participants who had been discharged from a secondary, tertiary, or medical school hospital and admitted to Khlong Luang Hospital (a community hospital) to continuously receive parenteral antimicrobial injection. Patients who were referred to another hospital or who died within 24 h of admission were excluded. This study comprised two phases: Phase 1, the pre-implementation group, constituted a previous study, as described elsewhere [12]; patients received standard treatment and data were collected from inpatient medical records and electronic databases over the first 6 months of the study (pre-implementation). Phase 2, the post-implementation group, consisted of a prospective implementation period during which patients received care following clinical practice guidelines (CPG) that had been established and approved by physicians, infectious disease pharmacists, and clinical pharmacists.

This study was approved by the Ethics Committee on Medical and Public Health Studies, Pathum Thani Provincial Public Health Office (PPHO-REC 2563/030). The ethics application for this study contained a waiver of consent because the participants would not be exposed to an increased risk of harm, and the clinical and adverse drug monitoring performed routinely by a laboratory was judged to be a quality improvement for the care of patients with sepsis in a community hospital.

### 2.2. Community Hospital-Based Parenteral Anti-Infective Therapy (CohPAT) Implementation

The CPG of CohPAT was established by physicians, infectious disease pharmacists, and clinical pharmacists, and approved by the Pharmaceutical and Therapeutic Committee in our hospital. This CohPAT was implemented and operated by a clinical pharmacist to (1) monitor the efficacy and safety of CohPAT as determined via laboratory measurements for white blood cell (WBC) count, renal function, and liver function, which were monitored at baseline and followed up during the OPAT in our setting; (2) monitor for adverse drug events during CohPAT; (3) adjust for antimicrobial dosing based on a parent’s renal function or liver function; (4) monitor causative pathogens and antimicrobial susceptibility testing (AST) from the original hospital if an initial finding was not reported, to select appropriate medication according to the culture and susceptibility results; (5) coordinate with the referring hospital if the pathogens revealed resistance to the antimicrobial drugs selected by the original hospital, and refer the patient back or ask the referring hospital to provide unavailable antimicrobials to administer in the referral setting, and (6) consult with a physician to refer a patient back to the referring hospital if the patient worsened clinically or if more medical investigations were needed.

### 2.3. Outcome Measurements

#### Process and Outcome Measurements

The study patients received a diagnosis for infection according to the Centers for Disease Control and Prevention/National Healthcare Safety Network (CDC/NHSN) Surveillance Definitions for Specific Types of Infections [14].

The data of the included participants in this study were gathered from medical records, and included the following: sex, age, comorbidities such as malignant tumor, hematologic malignancy, chronic kidney disease, chronic liver function disease, diabetes, neutropenia (defined as neutrophil and band cell count <500 cells/mL), connective tissue disease, or cardiovascular disease, the duration of admission, the sites of infections (based on CDC/NHSN), and antibiotic regimens.

In terms of clinical outcomes, in-hospital mortality was defined as being death during the hospital stay. A favorable treatment outcome was defined as being a composite of non-inhospital death and/or resolved or improved signs and symptoms of infections during the parenteral antimicrobial therapy in our setting. Contrarily, treatment failure (worsening signs and symptoms of infections) or in-hospital mortality were defined as being an unfavorable outcome. Clinical signs of infection and systemic inflammatory response syndrome items included body temperature >38 °C or <36 °C, respiratory rate >20 breaths per minute, heart rate >90 beats per minute, or leukocytosis <4000 or >12,000 cells/mL [15].

The duration of hospitalization was collected from the data in our setting from admission until discharge, death, or referral to the original hospital. The duration of antimicrobial use began from the date of the community hospital admission until discharge, death, or referral to the original hospital.

Data on the antimicrobial administration included the indication, dosage, administration, and the duration of antimicrobial use. The outcomes of pharmacist recommendations were considered to be “accepted” if any suggestion that was recommended by a pharmacist, including changing of the antimicrobials, adjustments to the antimicrobial dosage, or requests for essential laboratory testing more frequently than requests within routine practice were accepted by the physicians.

**Figure 1:**
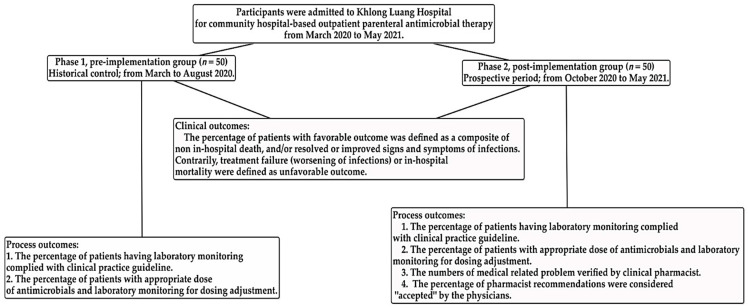
Diagram of study participation of analysis process and clinical outcomes.

Patient laboratory testing was performed and recorded at least once while the patient was in our setting, to monitor the efficacy, safety, and antimicrobial dosage adjustment (Figure 1).

### 2.4. Statistical Analysis

The study was designed on the basis of clinical outcomes among patients with OPAT in two related studies, to enroll the minimum number of participants per group [16,17]. The average proportion of a clinical cure of 0.85 (p), a precision value of 0.1 (d), and Z_α/2_ = 1.96 were used to determine the sample size. The number of participants in the pre- and the post-implementation groups was 50 per group. The descriptive statistics are presented as the percentages, the mean ± SD, and the median with interquartile range (IQR). A comparison of the outcomes between standard practice and intervention were made using a Chi-squared test or Fisher’s exact test, as appropriate, if the data had expected frequencies <5 with >20% of the cells. An independent *t*-test was performed to compare continuous variables with a normal distribution. The Mann–Whitney *U*-test was performed for data with a non-normal distribution. For unfavorable outcomes prediction, all significant variables in the univariate analysis were considered for the logistic regression analysis, and the two-sided significance level was set to 0.05. All analyses were performed using SPSS statistical software (IBM SPSS Statistics for Windows, IBM Corp., Armonk, NY, USA). The diagram of study participation was designed using CmapTools [18,19].

## 3. Results

During the study period of 14 months, 50 patients participated in the pre-implementation phase (Phase 1; 6 months), and 50 patients participated in the post-implementation phase (Phase 2; 8 months). The baseline characteristics of the participants are shown in Table 1. The median (IQR) age of the patients was 63 years (20) in Phase 1, and 66.5 years (18) in Phase 2. In Phases 1 and 2, 38 and 48% were men, respectively. The median (IQR) duration of treatment with antimicrobial parenteral injection in the community hospital was 6 (5) days in Phase 1 and 6.5 (4) days in Phase 2.

The length of hospital stay and the number of comorbidities were similar in both groups. However, for comorbidities, only cardiovascular diseases were significantly more frequent in Phase 1 than in Phase 2. The most frequent infectious diseases were lower respiratory infection (54%) in Phase 1 and bloodstream infection (38%) in Phase 2. Lower respiratory infection was significantly present in Phase 1, whereas skin and soft tissue were the dominant sites of infection in Phase 2 compared to in Phase 1 (Table 1).

Typically, the types of causative bacteria were similar in both groups. However, more *E. coli* isolates were found in Phase 2 than in Phase 1, whereas fewer *K. pneumoniae* isolates were found in Phase 2 (Table 1). A list of causative pathogens can be found in the Supplementary Material.

### 3.1. Process and Clinical Outcomes of Implementation

Laboratory monitoring complying with CPG was performed for a significantly higher number of patients in the post-implementation group than in the pre-implementation group (100% vs. 60%; *p* = 0.000) (Table 2). For 20 cases (40%) of non-compliance with CPG in the pre-implementation group, 16 cases did not receive laboratory monitoring either at baseline or follow-up period, whereas 4 cases were not monitored during CohPAT (Table 2).

During the post-implementation period, the results showed an increasing rate of appropriate antimicrobial dose received according to the guidelines, relative to the rate in the pre-implementation period (100% vs. 78%; *p* = 0.000). Among 11 cases with inappropriate doses or nonlaboratory monitoring in Phase 1, 7 (14%) out of 50 patients received an inappropriate antimicrobial dosage, and 4 (8%) out of 50 patients were not monitored for dose adjustment, especially for kidney function. Otherwise, all patients in Phase 2 received appropriate dosages of antimicrobial agents, increasing significantly from 78% in Phase 1 to 100% in Phase 2 (*p* = 0.004). Additionally, a significantly higher percentage of patients were found with unfavorable outcomes of antimicrobial treatment in Phase 1 (26%) than in Phase 2 (6%) (*p* = 0.006) (Table 2).

Regarding the univariate analysis, unfavorable outcomes included lower respiratory infection. Moreover, the unfavorable outcome rate was higher among the patients in Phase 1. Concerning the logistic regression analysis and the lower respiratory infection (adjusted OR, aOR 3.68; 95%CI 1.13–12.06), the post-implementation period (aOR 0.21; 95%CI 0.06–0.83) revealed significant risk factors that were associated with an unfavorable outcome (Table 3).

### 3.2. Description of Pharmacist Intervention

During the post-implementation period, all patients were monitored according to the practice guidelines, and the pharmacists reviewed four patients and asked for the results of bacterial culture tests and antimicrobial susceptibility testing from the referring hospitals. Eight medical problems were found: three improper dosages, three missing laboratory monitoring cases, one inappropriate therapy, and one maculopapular rash and itching case after using colistin. In the pharmacist consultations regarding the medical problems, all of the physicians accepted the respective pharmacist’s interventions.

## 4. Discussion

Theoretically, OPAT is the administration of intravenous antibiotics outside of the acute care hospital setting without intervening hospitalization. However, OPAT is an alternative to inpatient care administered in various settings, including at infusion centers, at home with health care professional services or caregiver(s), or in skilled nursing facilities [13]. A community hospital is a primary hospital, and more than 700 community hospitals are located across Thailand. Because community hospitals are close to the patient residences, they improve patient adherence and the ease of patient care for caregiver(s) in long-term usage of antimicrobials [20,21].

This is the first study in Thailand to highlight the important role of pharmacists for the health care of infectious patients continuously receiving antimicrobials in CohPAT. In a related study, Thomnoi and Santimaleeworagun stated that community hospitals could potentially be infusion centers for referring hospitals, such as university hospitals and regional center hospitals. However, collaboration on microbiology and coordination between health care settings, safety monitoring, and dosage adjustment were performed for each patient [12]. Thus, the CPG in CohPAT has to be established to improve the standard of care over the entire course of intravenous antimicrobials.

In this study, a higher percentage was observed for renal function monitoring among patients following CPG than among those who were in the pre-implementation group. Nonetheless, all patients in the post-implementation group received appropriate dosages of antimicrobial agents based on the monitoring of renal function in all participants. This important issue of laboratory monitoring during OPAT had been previously demonstrated. Huck et al. found that the recommended monitoring of laboratory test results, defined as the test results available for ≥1 week, e.g., complete blood counts, serum creatinine or liver function tests, varied with the antimicrobial therapy prescribed, and was significantly associated with reduced re-admissions while on OPAT [22]. Practically, British OPAT guidelines also recommended laboratory testing such as complete blood count with differential, kidney, and liver function, as well as other essential tests based on infectious diseases or specific antimicrobials [23]. Therefore, laboratory monitoring related to antimicrobials and dosage adjustment should be part of the care protocol [24].

The percentage of unfavorable treatment outcomes reduced from 26% in the pre-implementation period to 6% in the post-implementation period. The effective rate of CohPAT post-implementation was nearly the same as that in another study that evaluated effectiveness and safety for patients receiving parenteral injection, in a study by Suleyman et al., with a cure rate of 93%, with no patient remission within 30 days [16]. Unfortunately, our study did not evaluate the re-admission rate among participants receiving CohPAT. This patient outcome was an important finding for patients receiving OPAT, especially for infections caused by resistant pathogens, as in our study. Allison et al. reported that resistant organisms were significantly associated with 30-day re-admissions for patients receiving OPAT [25]. Moreover, the economic burden of CohPAT is an interesting issue. Interventions by an ID pharmacist to review OPAT care plans before hospital discharge were made for safety, efficacy, and cost savings in a related study by Heintz et al. [26]. Therefore, the re-admission and economic burden of CohPAT requires a study to determine the benefits of CohPAT for its more general use in real-world practice.

Pharmacists, as members of an ASP team, can improve antimicrobial use and patient care [13]. According to The American Society of Health-System Pharmacists (ASHP) guidelines, pharmacists should participate in the process of OPAT patient care, which includes reviewing patient laboratory results, checking drug interactions, monitoring renal function, and following up on patient allergies [27]. In our findings, pharmacists intervened in the medication management in decisions regarding appropriate indications, dosage adjustment, and adverse drug-reaction monitoring. Unfortunately, we did not use activity of therapeutic drug monitoring, due to unavailable drug concentration assays in our setting. This important activity could be supported by pharmacists for patients with OPAT and the ASP team, according to recommendations from the recent British OPAT guidelines [23].

Moreover, in the present study, antimicrobials such as carbapenems and colistin were continuously prescribed among patients receiving CohPAT. These antimicrobials were not available in the essential antimicrobial list of the community hospital. The pharmacists collaborating with nurses, had to ensure that the prescribed antimicrobials were administered in a safe condition according to the standard practice for infusion [12,22,26]. Therefore, a parenteral injection guideline should be adapted to use in a community hospital.

All medical problems related to antimicrobials were discussed in timely consultations with clinicians. Similarly, a related study by Shah et al. involving the monitoring of an OPAT guideline at a community hospital infusion unit found a significant difference in the adherence to the OPAT guidance between pharmacists and noninfectious disease physicians (100% vs. 35.9%, respectively) [21]. Therefore, pharmacists play an important role in persuading patients and clinicians to examine and correct a prescription so that a parenteral antimicrobial injection plan is effective and safe.

Among the patients with different infectious diseases, causative pathogens and resistant patterns who were referred to a community hospital for continuous treatment of parenteral injections between pre- and post-implementation. The type of bacteria and sites of infection, which were identified as predictors of mortality in a related study [28], had been herein analyzed by using logistic regression. The result showed that only lower respiratory infection was revealed as a risk of unfavorable outcome. It was similar to the result of a previous study by Vardakas et al. [28].

Several study limitations were encountered that should be considered. This study did not collect a sufficient amount of sample data to evaluate the other important factors that could have affected clinical outcomes, including patient severity, infection with antimicrobial-resistant pathogens, patient co-morbidities, and the type of infectious disease [28]. Aside from unfavorable outcomes among patients during the CohPAT program, the re-admission rate and the economic outcome were not assessed in the present study, which constitutes an important consequence, and could be found in OPAT management [29]. The variation of patients in their treatment, and the knowledge and attitude of physicians between the pre- and post-implementation groups might have differed, and could be confounders in our findings [30]. Additionally, the causative pathogens between two groups seemed to differ; e.g., *K. pneumoniae* caused more severe infections [31]. Future multicenter studies with a large number of participants would be useful to confirm the benefits of CohPAT.

## 5. Conclusions

Pharmacist-led CohPAT, as an antimicrobial treatment strategy for patients with infections, was applied in Thailand. An implementation of this treatment approach improved the quality of antimicrobial use and patient outcomes. We recommend that hospital policies consider adopting this treatment approach with the roles of pharmacists to improve treatment outcomes among patients, especially to provide seamless care when patients transition from one hospital to another.

## Figures and Tables

**Table 1:** Baseline characteristics of the patients and classification of antimicrobial therapy in the pre-implementation and post-implementation periods.

Characteristic	Pre-Implementation (*n* = 50 Cases)	Post-Implementation (*n* = 50 Cases)	*p*-Value
Age—years (median; IQR)	63 (20)	66.5 (18)	0.603 ^a^
Female sex—no. (%)	19 (38)	24 (48)	0.313 ^b^
Length of stay in community hospital—days (median; IQR)	7.5 (7)	7.5 (9)	0.663 ^a^
Antimicrobial treatment duration in community hospital—days (median; IQR)	6 (5)	6.5 (4)	0.642 ^a^
Number of comorbidities—no. (%)			
No underlying disease	8 (16)	3 (6)	0.227 ^b^
1 disease	18 (36)	12 (24)	
2 diseases	12 (24)	18 (36)	
3 diseases	7 (14)	11 (22)	
4 diseases	5 (10)	6 (12)	
Comorbidities—no. (%)			
Diabetes mellitus	14 (28)	17 (34)	0.517 ^b^
Malignancy	7 (14)	8 (16)	0.779 ^b^
Chronic kidney disease	8 (16)	6 (12)	0.564 ^b^
Cardiovascular disease	25 (50)	4 (8)	0.000 ^b^
Cerebrovascular disease	6 (12)	10 (20)	0.275 ^b^
Chronic lung disease	2 (4)	3 (6)	1.000 ^a^
Liver disease	1 (2)	1 (2)	1.000 ^a^
Referring hospital—no. (%)			
Provincial hospital	31 (62)	35 (70)	0.616 ^b^
Medical school	16 (32)	9 (18)	
Others	3 (6)	6 (12)	
Site of infection—no. (%)			
Lower respiratory	27 (54)	15 (30)	0.015 ^a^
Bloodstream	13 (26)	19 (38)	0.198 ^a^
Urinary tract	7 (14)	13 (26)	0.134 ^a^
Intra-abdomen	5 (10)	5 (10)	1 ^a^
Skin and soft tissue	3 (6)	11 (22)	0.021 ^a^
Osteoarticular	3 (6)	1 (2)	0.617 ^c^
Central nervous system	1 (2)	1 (2)	1 ^c^
Cardiovascular system	1 (2)	0 (0)	1 ^c^
Causative bacteria—no. (%)			
*E.* *coli*	1 (2)	11 (22)	0.002 ^b^
*K. pneumoniae*	7 (14)	2 (4)	0.160 ^c^
*P. aeruginosa*	2 (4)	3 (6)	1.000 ^c^
*A. baumannii*	5 (10)	6 (12)	0.749 ^b^
*S. aureus*	2 (4)	3 (6)	1.000 ^c^
Infection with antimicrobial resistant bacteria—no. (%)			
*K. pneumoniae* (MDR)	3 (6)	0 (0)	0.092 ^c^
*E. coli* (MDR)	1 (2)	9 (18)	0.014 ^c^
*A. baumannii* (CRAB)	4 (8)	4 (8)	1 ^c^
*S. aureus* (MRSA)	1 (2)	1 (2)	1 ^c^

^a^ Mann–Whitney *U*-test; ^b^ Pearson Chi-squared test; ^c^ Fisher’s exact test. Abbreviations: CRAB, carbapenem-resistant *A. baumannii*; MDR, multidrug resistance (resistant to at least 3 classes of antimicrobials); IQR, interquartile range; MRSA, methicillin-resistant *S. aureus.*

**Table 2:** Process and clinical outcomes of implementation in the pre-implementation and post-implementation periods.

Outcome	Pre-Implementation (*n* = 50 Cases)	Post-Implementation (*n* = 50 Cases)	*p*-Value
Dose adjustment and laboratory monitoring—no. (%)			
Appropriate dose of antimicrobials	39 (78)	50 (100)	0.000
Inappropriate dose of antimicrobials or non-laboratory monitoring for dose adjustment	11 (22)	0 (0)	
Laboratory monitoring * complied with CPG—no. (%)			
Compliance with CPG	30 (60)	50 (100)	0.000
Non-compliance with CPG	20 (40)	0 (0)	
Clinical outcomes—no. (%)			
Favorable outcome	37 (74)	47 (94)	0.006
Unfavorable outcomes	13 (26)	3 (6)	
Death	4	0	
Treatment failure	9	3	

* Laboratory monitoring of white blood cell count, renal function, and liver function. Abbreviations: CPG, clinical practice guidelines.

**Table 3:** Factors predicting unfavorable outcome among patients with CohPAT using univariate analysis and logistic regression analysis.

Characteristic	Unfavorable Outcome (*n* = 16 Cases)	FavorableOutcome (*n* = 84 Cases)	OR (95% CI)	aOR (95% CI)
Age ≥60 years—no. (%)	11 (68.8)	52 (61.9)	1.35 (0.43–4.26)	
Female sex—no. (%)	6 (37.5)	37 (44)	0.76 (0.25–2.29)	
Comorbidities ≥3 diseases—no. (%)	3 (18.8)	26 (31)	0.52 (0.14–1.96)	
Comorbidities—no. (%)				
Diabetes mellitus	2 (12.5)	29 (34.5)	0.27 (0.06–1.27)	
Malignancy	3 (18.8)	12 (14.3)	1.39 (0.34–5.59)	
Chronic kidney disease	1 (6.3)	13 (15.5)	0.36 (0.04–3.00)	
Cardiovascular disease	6 (37.5)	23 (27.4)	1.59 (0.52–4.88)	
Cerebrovascular disease	1 (6.3)	15 (17.9)	0.31 (0.04–2.50)	
Chronic lung disease	1 (6.3)	4 (4.8)	1.33 (0.14–12.77)	
Liver disease	1 (6.3)	1 (1.2)	5.53 (0.33–93.37)	
Site of infections—no. (%)				
Lower respiratory tract	11 (68.8)	28 (33.3)	4.4 (1.39–13.9)	3.68 (1.13–12.06)
Bloodstream	4 (25)	26 (31)	0.74 (0.22–2.53)	
Urinary tract	0 (0)	20 (23.8)	0.10 (0.01–1.66) ^a^	
Intra-abdomen	2 (12.5)	8 (9.5)	1.36 (0.26–7.07)	
Skin and soft tissue	2 (12.5)	13 (15.5)	0.78 (0.16–3.85)	
Causative bacteria—no. (%)				
*E. coli*	0 (0)	12 (14.3)	0.18 (0.01–3.12) ^a^	
*K. pneumoniae*	2 (12.5)	7 (8.3)	1.57 (0.30–8.36)	
*P. aeruginosa*	0 (0)	5 (6)	0.44 (0.02–8.31) ^a^	
*A. baumannii*	13 (18.8)	8 (9.5)	2.19 (0.51–9.36)	
*S. maltophilia*	1 (6.3)	0 (0)	16.35 (0.64–420.18) ^a^	
*S. aureus*	0 (0)	5 (6)	0.44 (0.02–8.31) ^a^	
Infection with antimicrobial resistant bacteria—no. (%)				
*E. coli* (MDR)	0 (0)	10 (11.9)	0.22 (0.01–3.86) ^a^	
*K. pneumoniae* (MDR)	1 (6.3)	2 (2.4)	2.73 (0.23–32.08)	
*A. baumannii* (CRAB)	2 (12.5)	6 (7.1)	1.86 (0.34–10.15)	
*S. aureus* (MRSA)	0 (0)	2 (2.4)	1.00 (0.05–21.80) ^a^	
Post-implementation period	3 (18.8)	47 (56)	0.18 (0.05–0.67)	0.21 (0.06–0.83)

^a^ Haldane correction; Abbreviations: CRAB, carbapenem-resistant *A. baumannii*; MDR, multidrug resistance (resistant to at least 3 classes of antimicrobials); IQR, interquartile range; MRSA, methicillin-resistant *S. aureus*.

## Data Availability

Not applicable.

## Supplementary Materials

The following are available online at https://www.mdpi.com/article/10.3390/antibiotics11060760/s1, Table S1: The causative organisms in the pre-implementation and post-implementation groups.
