# Supplementary material for: Impact of Pharmacist-Led Implementation of a Community Hospital-Based Outpatient Parenteral Antimicrobial Therapy on Clinical Outcomes in Thailand
Source: Antibiotics (Basel). 2022 Jun 2;11(6):760. doi: 10.3390/antibiotics11060760 (PMC9220076; doi:10.3390/antibiotics11060760)
Supplement: Supplementary file 1 [file antibiotics-11-00760-s001.zip › antibiotics-1620714-supplementary.pdf]

Supplementary Materials

Table S1. The causative organisms in the pre-implementation and post-implementation groups.

Pathogens	Pre-implementation	Post-implementation
Bacterial culture — no. (%)	(*n* = 50 participants)	(*n* = 50 participants)
Patients with culture negative status	26 (52)	22 (44)
Patients with culture positive status	24 (48)	28 (56)
Gram strain — no. (%)	(*n* = 26 isolates)	(*n* = 32 isolates)
Gram negative bacteria	22 (84.6)	27 (84.4)
Gram positive bacteria	4 (15.4)	5 (15.6)
Causative organisms — no. (%)	(*n* = 26 isolates)	(*n* = 32 isolates)
Escherichia coli	1 (3.8)	11 (34.4)
Klebsiella pneumoniae	7 (26.9)	2 (6.3)
Klebsiella ozonae	1 (3.8)	1 (3.1)
Acinetobacter baumannii	5 (19.2)	6 (18.8)
Acinetobacter lwoffii	0 (0)	2 (6.3)
Pseudomonas aeruginosa	2 (7.7)	3 (9.4)
Stenotrophomonas maltophilia	2 (7.7)	0 (0)
Acromobacter spp.	2 (7.7)	0 (0)
Proteus spp.	2 (7.7)	1 (3.1)
Enterobacter cloacae	0 (0)	1 (3.1)
Staphylococcus aureus	2 (7.7)	3 (9.4)
Staphylococcus haemolyticus	1 (3.8)	1 (3.1)
Viridans group streptococci	0 (0)	1 (3.1)
Enterococcus faecalis	1 (3.8)	0 (0)

Abbreviations: CRAB, carbapenem-resistant *A. baumannii*; ESBL, extended-spectrum beta-lactamase; MDR, multidrug resistance; MRSA, methicillin-resistant *S. aureus*; MSSA, methicillin-susceptible *S. aureus.*
